# Fibrx Rocking Chair: Design and Application of Tailored Timber as an Embedded Frame for Natural Fibre-Reinforced Polymer (NFRP) Coreless Winding

**DOI:** 10.3390/polym15030495

**Published:** 2023-01-18

**Authors:** Alexandra Pittiglio, Ailey Simpson, Vanessa Costalonga Martins, Hanaa Dahy

**Affiliations:** 1 ITECH Master Program, University of Stuttgart, Keplerstr. 11, 70174 Stuttgart, Germany; 2 BioMat@Stuttgart: Bio-Based Materials and Materials Cycles in Architecture, Institute of Building Structures and Structural Design, University of Stuttgart, Keplerstr. 11, 70174 Stuttgart, Germany; 3 BioMat@Copenhagen: Bio-Based Materials and Materials Cycles in the Building Industry Research Centre-TECH-Technical Faculty for IT & Design, Planning Department, Aalborg University, Meyersvænge 15, 2450 Copenhagen, Denmark; 4 Department of Architecture (FEDA), Faculty of Engineering, Ain Shams University, Cairo 11517, Egypt

**Keywords:** natural fibre-reinforced polymers, NFRP, coreless filament winding, CFW, hybrid material system, computational design, lightweight structure

## Abstract

The building industry needs to innovate towards a more sustainable future and can do so through a combination of more renewable material choices and less wasteful fabrication processes. To address these issues, a hybrid material and fabrication system was developed using laminated timber veneer and natural fibre-reinforced composites (NFRPs), two materials that are leveraged for their potential of strategic material placement in additive processes towards programmed material behaviour and performance. The main contribution is in the hybrid fabrication approach, using thin, bent laminated veneer as an embedded frame for coreless filament winding of NFRP, which removes the need for temporary, wasteful formwork that is typically required to achieve structurally performative bent timber or FRP elements. Integrative methods are developed for the design, simulation, and fabrication of a rocking chair prototype that illustrates the architectural potential of the developed fabrication approach.

## 1. Introduction

Consuming 50% of all extracted materials throughout the European Union and accounting for 35% of greenhouse gases (GHGs), it has been widely acknowledged that the building industry must innovate toward a more sustainable future [1]. To address this, efforts have largely been centred around improving the operational efficiency of occupied buildings in terms of their energy consumption. However, there is substantial evidence pointing to the significance of embodied carbon in contributing to emissions, meaning the GHGs resulting from the processing of building materials and the construction process [2]. Decreasing the embodied carbon of buildings can be realised through a combination of more renewable material choices, more efficient use of materials, and less wasteful fabrication processes.

**Figure 1:**
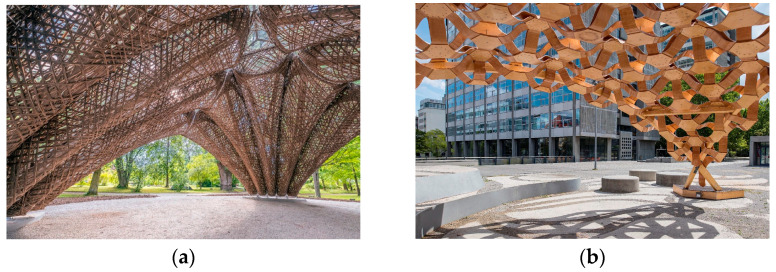
State-of-the-art projects that apply NFRP to structural components: (**a**) LivMatS–ICD/ITKE Research Pavilion (2020–2021). Flax fibre–polymer composite used in segmented shell structure [15]; (**b**) BioMat Pavilion 2018. Segmented shell structure made from lignocellulosic flexible core reinforced by timber veneer layers (vacuum-formed) [9].

The high strength-to-weight ratio and anisotropic properties of fibre-reinforced polymers (FRPs) have made them suitable for use in high-performance applications, with widespread use in the aerospace and automotive industries [3]. Over the last decade, research projects completed by the Institute for Computational Design (ICD), the Institute of Building Structures and Structural Design (ITKE), and the Department of Bio-Based Materials and Materials Cycles in Architecture (BioMat) at the University of Stuttgart have demonstrated the applicability of FRPs towards architectural elements [4,5,6,7,8,9,10,11,12,13,14,15] (Figure 1). The combination of filaments with a hardening matrix, such as epoxy resin, allows the fibres to not only withstand tensile loads but also to handle significant compressive loads in long-span, load-bearing, composite structures [12]. In addition, with a lower carbon footprint, fibres of organic origin, such as flax or hemp, present renewable alternatives to their synthetic counterparts [16,17,18].

**Figure 2:**
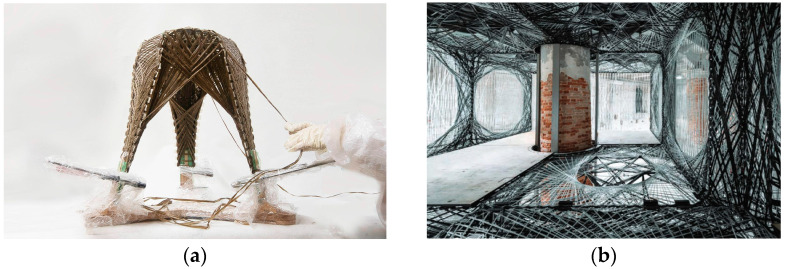
State of the art: (**a**) FlexFlax stool embedded winding frame [13]; (**b**) hybrid timber–fibre slab of the Maison Fibre [11].

In automotive and aerospace industries, FRP fabrication processes typically rely on customised moulds for the mass production of standardised elements, a process inapplicable in architecture, where highly differentiated components are required [3]. Coreless filament winding (CFW), a novel additive fabrication process, tries to overcome this limitation by eliminating the mould in the fabrication process. However, CFW still relies on large bespoke frames that are project-specific and usually cannot be easily reconfigured [4,5]. Previous state-of-the-art projects have tried to address this shortcoming by embedding the winding frame into their final components [13,19,20]. Of note is the *FlexFlax* stool (Figure 2a), which uses a tailored flax fibre preform as an embedded winding frame, simplifying the NFRP winding process and resulting in a lightweight stool that can carry 80 times its weight [13].

**Figure 3:**
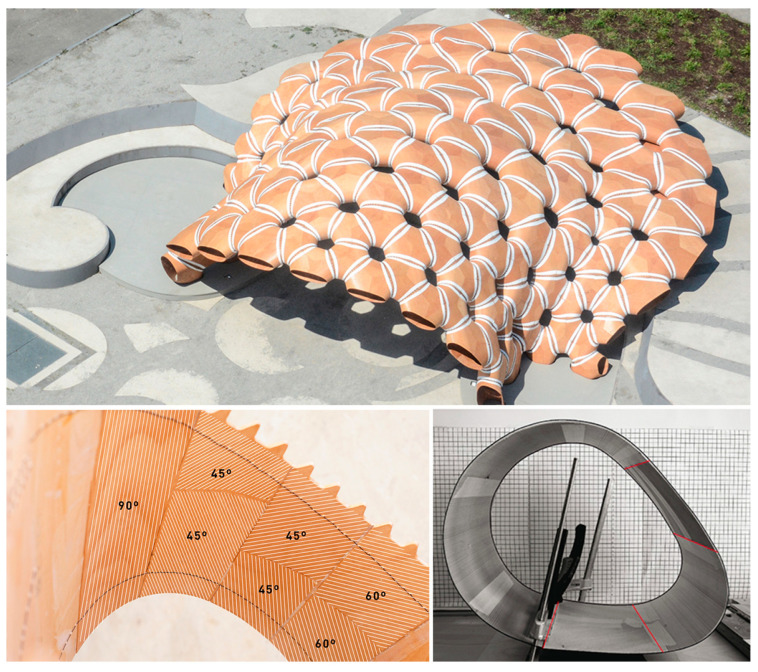
ICD/ITKE Research Pavilion 2015-16: Segmented timber shell and custom lamination of timber veneer to achieve specific bending and strength requirements of architectural shell elements [22].

Other projects have explored the integration of a conventional off-the-shelf material to simplify CFW fabrication. This is the case of the slab component in the *Maison Fibre* (Figure 2b) [11]. The fibres are here hybridised with timber, which not only contributes to the overall structural stability but also lends extra functionality to the system, serving as a trafficable surface, a condition that is hard to achieve with CFW alone [11,21]. Similar to FRPs, timber is also an anisotropic building material. However, standard processing aims to produce more predictable, orthotropic timber building material, with grain direction following a single axis. Previous timber research projects have pointed to the form and functional potential of leveraging these anisotropic properties. The ICD/ITKE Research Pavilion 2015-16 utilised strategic grain directionality through timber veneer lamination, tailoring bending, and strength behaviour to achieve the desired function and performance of the fabricated building modules (Figure 3) [22]. Using the strategic lamination of veneer towards programmed bending allows for the design of performative elements in an additive fabrication process, as opposed to the traditional methods of achieving bent timber that include subtractive processes or intensive moulds.

The research presented in this paper aims to leverage the anisotropic qualities of both timber and NFRP through a hybrid material system. By embedding thin timber as a winding frame for CFW, the hybridisation potential is demonstrated not only in the advanced performance of the resulting element but also throughout the fabrication process. The research involved a bottom-up material investigation phase and development of the hybrid roles and interfaces, fabrication approach, and simulation workflow. This culminated in the production of a rocking chair, capable of supporting a dynamic human load. The chair can be viewed as an analogue for a larger structural system and fabrication method, capable of being applied to the architectural scale.

## 2. Materials and Methods

### 2.1. Materials

#### 2.1.1. Timber Veneer

**Figure 4:**
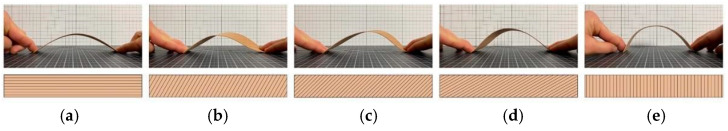
Bending studies with varied grain direction of thin timber veneer: (**a**) 0-degree primary grain direction; (**b**) 30 degrees; (**c**) 45 degrees; (**d**) 60 degrees; (**e**) 90 degrees.

Timber veneer is a thin, bendable, and sometimes brittle material. Its utilisation as a bent winding frame requires staying within its elastic deformation range, without permanently warping or snapping it. This involves understanding the bending behaviour, as grain direction varies with respect to the long axis of the element. Figure 4 illustrates how the primary grain direction impacts stiffness or resistance to bending. Along the spectrum ranging from 0 to 90 degrees of the primary grain direction, stiffness decreases as bending flexibility increases. Non-orthogonal grain directions (30, 45, and 60 degrees) result in torsional bending.

#### 2.1.2. Flax Fibre-Reinforced Composite

To produce the FRP, flax fibre was combined with epoxy resin through a process known as *fibre impregnation*. For this project, a simplified version of the drum-type resin bath used for the ICD/ITKE Buga Pavilion [10] was employed. Drum-type resin baths are commonly used in the filament winding industry, and their mechanisms are already described in detail in the literature [3,23,24]. The process of impregnation involved passing two input flax fibre rovings through a series of drums in a container filled with resin and then, through an eyelet that joined the two strands and removed excess resin, creating one single fibre bundle. However, whereas most winding processes use the resin bath for in situ winding, this research decided to fabricate pre-impregnated spools to minimise resin spilling during winding. The fibres were pre-impregnated prior to the fabrication stage by passing them through the resin bath into a bobbin and storing them at a low temperature of −10 °C, to slow curing. The pre-impregnation process allows for a controlled environment for the material preparation and ensures proper impregnation and more viscous consistency when winding the fibres to the final structure.

The flax fibre used was 100% flax fibre roving from Depestele (product code LinCore FR2400), of Tex 2400 (linear density). The same material was used in the research and material benchmarking of the LivMatS Pavilion [15]. Therefore, for this research, a composite system similar to the one used on the LivMatS Pavilion was produced. Synthetic epoxy resin was used (3 parts EPIKOTE Resin MGS RIMR 235 + 1 part EPIKURE Curing Agent RIMH 237, Pot Life: 48 h, all produced by Hexion Inc., Columbus, OH, USA) as the base matrix, due to availability and prior experience with the products. While bio-resins are available, the literature shows that they have worsened impregnation in comparison to cured epoxy resins, which could result in a higher environmental impact than natural fibres [15,25]. New material characterisation or tests of the microstructure were not performed, as the final composite was assumed to have similar mechanical properties as the flax composite in the LivMatS Pavilion [15]. It is important to note that for coreless filament winding, the fibre volume ratio (FVR) along the bundles can vary as a result of fabrication parameters, such as differences in tension during winding [15]. Therefore, based on the literature, the fibre mass ratio (FMR) was assumed to be around 0.33, and the FVR was around 0.28 [15]. The determination of exact FVR and FMR, like the mechanical characterisation of the composite, lay outside the scope of this project.

### 2.2. Methods

#### 2.2.1. Design Process

To understand how thin timber and FRPs could be combined in a material hybrid, an initial study was conducted that demonstrated how the composite system could achieve structural potential greater than the sum of its parts. A comparison of the material properties of timber and NFRP indicate that the two would be effectively paired in a structural scenario where timber could primarily handle compressive forces, and NFRP primarily undergoes tension. From a functional perspective, timber offers a surface quality to the material hybrid, capable of creating a comfortable seat. As a linear element, fibres can have sharp and uncomfortable edges when cured, making them more suitable as structural reinforcement, tucked into areas where a user would not make direct contact with them.

**Figure 5:**
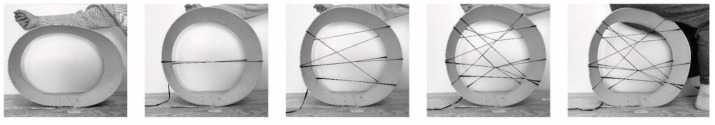
Initial study sequentially placing carbon FRP on a thin timber element, reinforcing most deformed regions.

Figure 5 depicts an early material hybridisation study, resembling a wheel-and-spoke element. The goal was to understand how FRP paired with timber impacted the performance of the resulting hybrid. This study involved incrementally adding fibres to a thin timber wheel, applying a consistent load to the top, documenting the most deformed regions, and further applying another increment of fibre as reinforcement. This continued until a strong hybrid wheel and spoke resulted, capable of handling a full human load without large deformations. This empirical study informed how these two materials could come together in a performative way. It indicated how few fibres would be required while still seeing impactful results, especially when fibres would be primarily used as tensile reinforcement, similar to that of a spoke. Furthermore, it indicated how thin the timber could be, even when used to support a human load, when tensile reinforcement was present.

**Figure 6:**
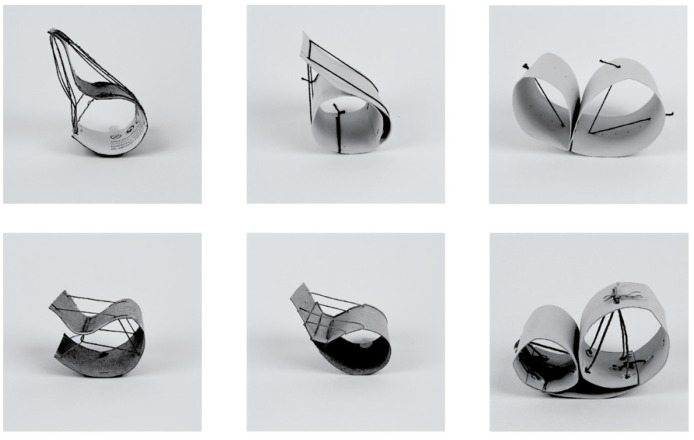
Paper–string prototypes demonstrating how timber (paper) could be used in compression, and flax FRP (string) in tension.

Paper–string models allowed for the quick prototyping of the two materials coming together in an abstracted global design, with paper and string representing thin timber and flax FRP, respectively (Figure 6). This process provided an understanding of the geometric possibilities of a single curved surface element and explored where fibres could be placed relative to the surface so that they would primarily act in tension when a sitting load was applied. An omega shape was selected for further processing since it makes use of the bending strength of the surface, there is a clear relationship between surface compression and fibres in tension, and it presented an opportunity to fabricate using a continuous timber strip rather than discrete timber elements.

**Figure 7:**
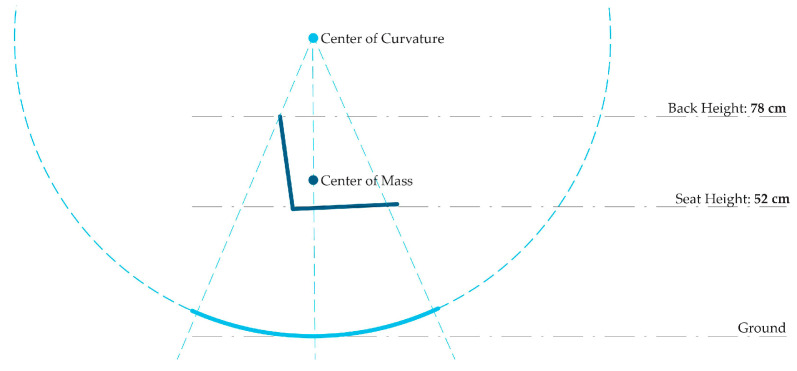
Curvature, ergonomics, and rocking chair functionality curve diagram.

With the understanding that a material hybrid comprising thin timber and FRP could be a performative and efficient use of materials, a formal design approach was developed by considering both bottom-up material investigation and top-down design constraints. From a design constraint perspective, the curvature and weight distribution of a rocking chair must be carefully considered (Figure 7). The centre of mass of the combined chair and person must align vertically with the centre of the curvature of the rocking surface. The curvature of the rocking surface should be a smooth arc with a defined radius. Knowing this, a bottom-up investigation could then inform the use of materials to achieve these design goals.

#### 2.2.2. Tailoring Timber Veneer

Moving away from paper prototyping and towards working with real materials, testing began that aimed to inform on the material behaviour of timber veneer. The design goal of creating a rocking chair meant that particular attention had to be paid to the consistency of bending across the bottom surface of the timber. Likewise, a comfortable sitting surface could only be achieved through locally specific bending at the seat region. Combining layers of timber veneer through lamination created an opportunity to locally vary the bending curvature and stiffness of the timber. The following tests were carried out to investigate the potential of custom lamination strategies.

##### Lamination Bending Test 1: Single Strip

**Figure 8:**
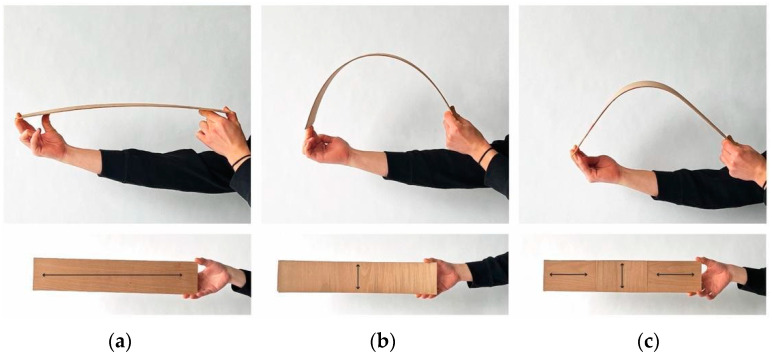
Bending studies with custom laminated grain directions: (**a**) 0 degrees with 0-degree bilayer; (**b**) 0 degrees with 90-degree bilayer; (**c**) mixed, 0 degrees with 0–90–0-degree bilayer.

Test 1 involved various lamination patterns, each with two layers of thin veneer. The first layer of veneer had a 0-degree grain direction and was consistent across all three studies. The second layer of veneer was where the pattern varied, with 0-degree, 90-degree, and mixed grain directions. Bending behaviour is visible in the following set of images in Figure 8.

##### Lamination Bending Test 2: Combined

**Figure 9:**
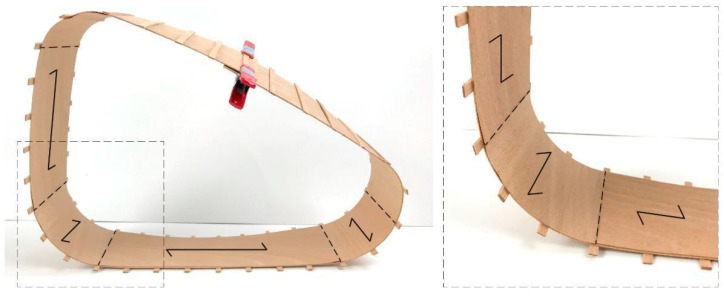
Combination of lamination and bending studies for the application of a chair. Grain direction is highlighted with black arrows.

Test 2 involved combining the single strip studies in *Test 1* to investigate a single bent surface element that began to demonstrate how these concepts may be applied to the overall form of a chair. As shown in Figure 9, higher curvature was achieved through a 0-degree with a 90-degree layup of timber veneer and applied in between the back, ground, and seat regions. Lower curvature and higher strength were achieved through a 0-degree with 0-degree layup of timber veneer and applied in the back, ground, and seat regions. In situ lamination was performed by applying glue, bending the laminated veneer into place, and applying clamps to fix the geometry while the glue was cured.

#### 2.2.3. Tailoring Flax FRP

**Figure 10:**
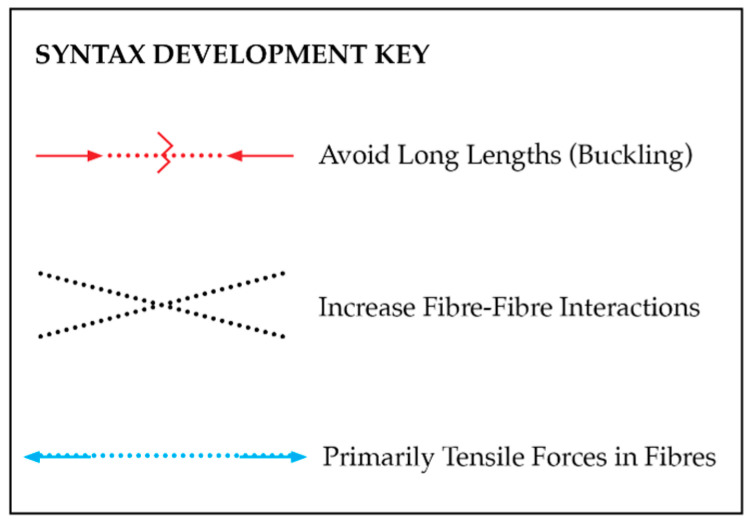
Fibre syntax development goals to avoid typical failure modes.

Before generating a fibre-winding syntax, which, in this study, is the term for the sequence in which the fibres are laid [12], general guidelines were considered based on precedent research. To achieve maximum performance and avoid buckling failure, coreless filament winding should aim to reduce fibre lengths, increase interactions, and place fibres primarily in tension. Typical failure models are buckling, fibre fracture, and delamination [15]. Figure 10 outlines the syntax development guidelines used to mitigate typical failure modes.

In terms of digital simulation, there are many challenges when trying to predict the behaviour and structural performance of the final syntax layup. As known, the final geometry and curvature of the wound element are connected to fibre interaction; therefore, it is hard to digitally predict the precise location of every layup [3,26]. In addition, the fibre volume ratio (FVR) is not fixed, as it depends on the impregnation and tension during the composite fabrication and the winding process [26]. This, combined with the anisotropy of the material, makes the system very complex to predict. Previous research points to the fact that it is not possible to use conventional stress-based design tools, and that the most complete methodology of simulation for coreless wound elements involves a combination of prototyping and material testing on both small and large scale, coupled with a digital structural feedback loop [26]. For this project, due to the smaller scale of the final chair prototype, a simplified version of this methodology was applied. As mentioned, literature values were used to inform the mechanical properties of the materials in the simulation, while both physical-scale models and full-scale prototypes were used to calibrate the fibre behaviour in the digital simulations.

**Figure 11:**
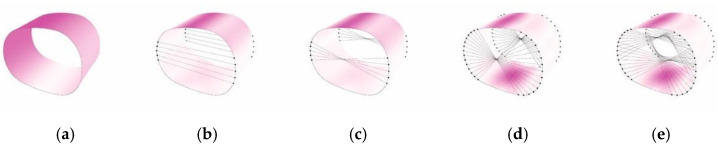
Timber surface deflection analysis: (**a**) no FRP winding applied; (**b**) in-plane winding, no fibre interaction; (**c**) in-plane cross-winding; (**d**) denser syntax, in-plane cross-winding; (**e**) alternating edge cross-winding with many points of fibre interaction.

The fibre layup was developed through an iterative feedback loop between syntax simulations and structural analysis using *Rhinoceros 3D, Grasshopper Computational Modelling Software*, and *Karamba3D*, a parametric FEM plugin for *Grasshopper*. Focusing specifically on the seat of the chair, where the highest loads would occur, the above strategies for syntax development were applied. Deflection analysis was applied to the timber surface to compare the effectiveness of different syntax approaches. The analysis sequence seen in Figure 11 begins with no fibre, and gradually a denser and more effective syntax is observed, resulting in lesser deformation, thus validating that using the FRP material with the principles described above can be successful.

**Figure 12:**
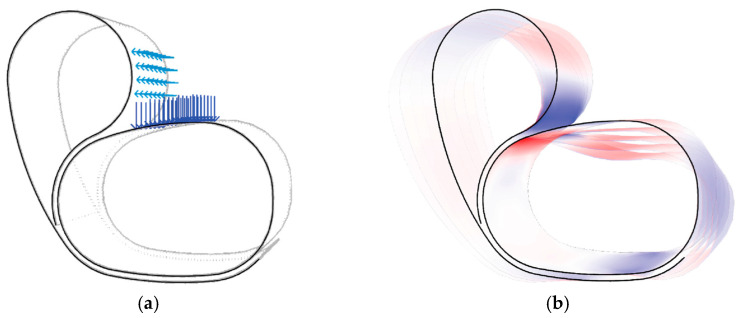
Deformation analysis informing syntax: (**a**) expected loading conditions of the chair, with 100 kg distributed load applied to the seat and an additional 30% applied to the back; (**b**) exaggerated deformation behaviour of the chair under applied loads.

The development of the full fibre syntax involved applying a distributed load to the seat surface of the chair equivalent to a 100 kg human, as well as applying 30% of this load towards the surface behind a user’s back. As shown in Figure 12a, the primary loading would occur in the region indicated by the blue force vectors. The primary force analysis, illustrated in Figure 12b, indicates regions of compression (red) and tension (blue) along the timber surface. Furthermore, visualising the deformation behaviour, shown in an exaggerated view, highlights the regions likely to bulge outward versus those likely to buckle inwards. This method was used to inform FRP material placement, aiming to provide tensile reinforcement to those regions likely to undergo surface tension.

**Figure 13:**
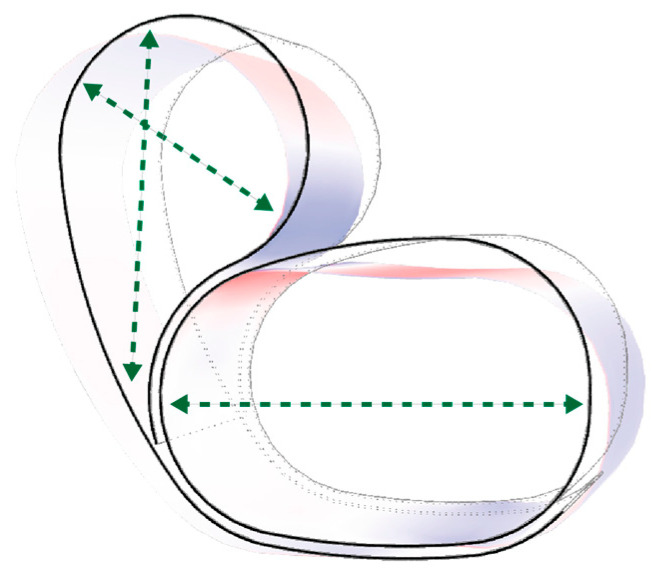
Tensile reinforcement regions that would prevent bulging deformation and high timber surface tensile forces.

The finite element analysis (FEA) simulations began by abstracting the timber element as a surface element and applying the expected loading conditions to the chair to determine the primary forces within the timber surface, as well as deformation behaviour. This analysis was used to identify the regions of the timber surface that would most benefit from tensile reinforcement. The green arrows spanning across Figure 13 highlight those regions in which the tensile reinforcement of flax FRP would mitigate the high-tension (blue) forces exerted on the unreinforced timber surface, and bulging deformation would be prevented.

**Figure 14:**
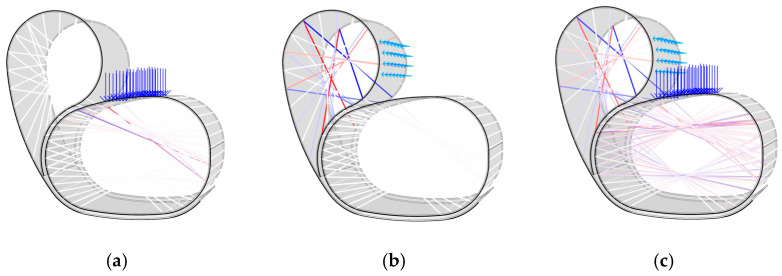
Fibre beam finite element analysis: (**a**) the seat of the chair; (**b**) the back of the chair; (**c**) the entire system working in cohesion.

Following the current state-of-the-art literature methodology for simulating coreless filament winding structural behaviour, an FEA beam analysis was performed to model the fibres in the system and determine the axial tensile and compressive forces [26,27]. Each bundle on the layup was considered to be a beam element between interaction points, while the timber element was modelled as a shell surface. Primary attention was paid to high compressive forces in the fibres since they are a risk of causing buckling failure, as seen in past FRP research projects and accompanying structural testing [15]. Figure 14 highlights these findings for the back of the chair, the seat, and the combined system working in cohesion. As mentioned, a 100 kg load was applied to the seat, shown in Figure 14a, while 30 kg (30% of the seat load) was applied to the back, as seen in Figure 14b. Using a fibre bundle diameter of 6 mm throughout the entire chair, the maximum compressive forces were determined to be −0.72 N in the seat and −0.33 N in the back region. Based on the axial compression tests from previous CFW flax FRP projects [15], these values were determined to be within a suitable range for this material.

#### 2.2.4. Timber and FRP Connections: Anchor Point Development

**Figure 15:**
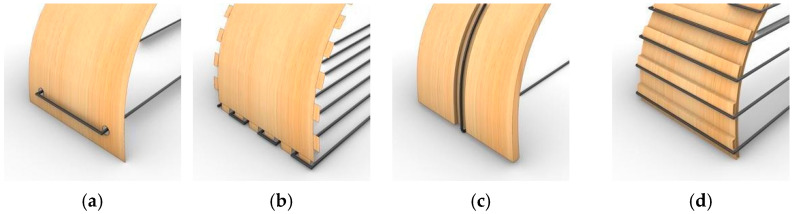
Development of the timber–fibre connections through subtractive methods at edges: (**a**) subtractive holes; (**b**) subtractive teeth; (**c**) subtractive long-edge grooves; (**d**) additive short-edge grooves.

Using thin timber veneer as an embedded FRP winding frame necessitated the design of integrated anchor points that would allow for CFW while maintaining the material integrity of the relatively fragile frame material, particularly at weak edge conditions. Using additive rather than subtractive methods was preferred, to strengthen the material in these critical connection zones and prevent material waste. Multiple connection strategies were tested involving subtractive fabrication at edge conditions, including holes, grooves, and teeth (Figure 15a–c). Upon testing, these methods all proved to negatively impact the material performance of the thin timber veneer, causing the shearing or ripping of the timber material when FRP winding was applied.

Additive short-edge grooves were developed to address the connection problems highlighted above (Figure 15d). The orthogonally placed grooves were built additively through lamination. Their orthogonal orientation prevented them from significantly impacting the bending behaviour of the timber while providing a channel for the fibre to sit against to secure its placement during fabrication.

#### 2.2.5. Fabrication

**Figure 16:**
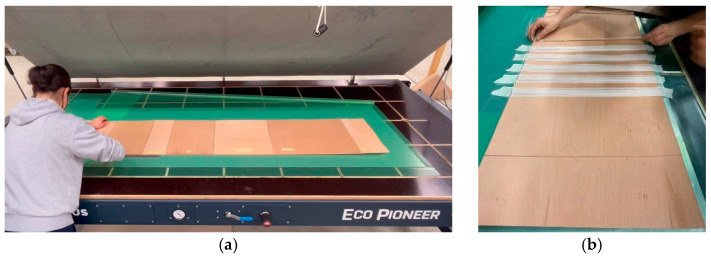
Vacuum table lamination of timber elements: (**a**) preparing timber surface lamination; (**b**) preparing additive groove lamination.

The fabrication process involved material preparation and assembly. The material preparation stage included preparing the pre-impregnated flax FRP and flat-laminated veneer. For the final prototype, a vacuum press was used for flat lamination to ensure the adequate adhesion of the veneer bilayer and additive teeth, as seen in Figure 16. 

**Figure 17:**
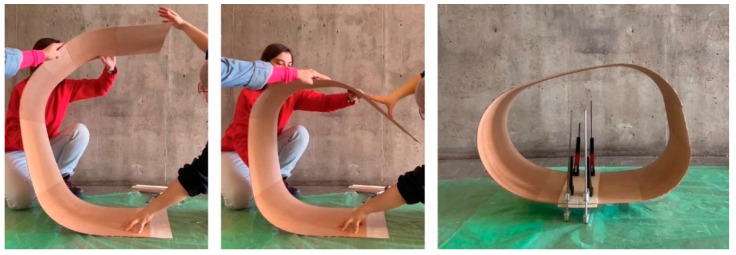
In situ timber lamination of the seat surface, to connect ends and achieve desired curved geometry.

**Figure 18:**
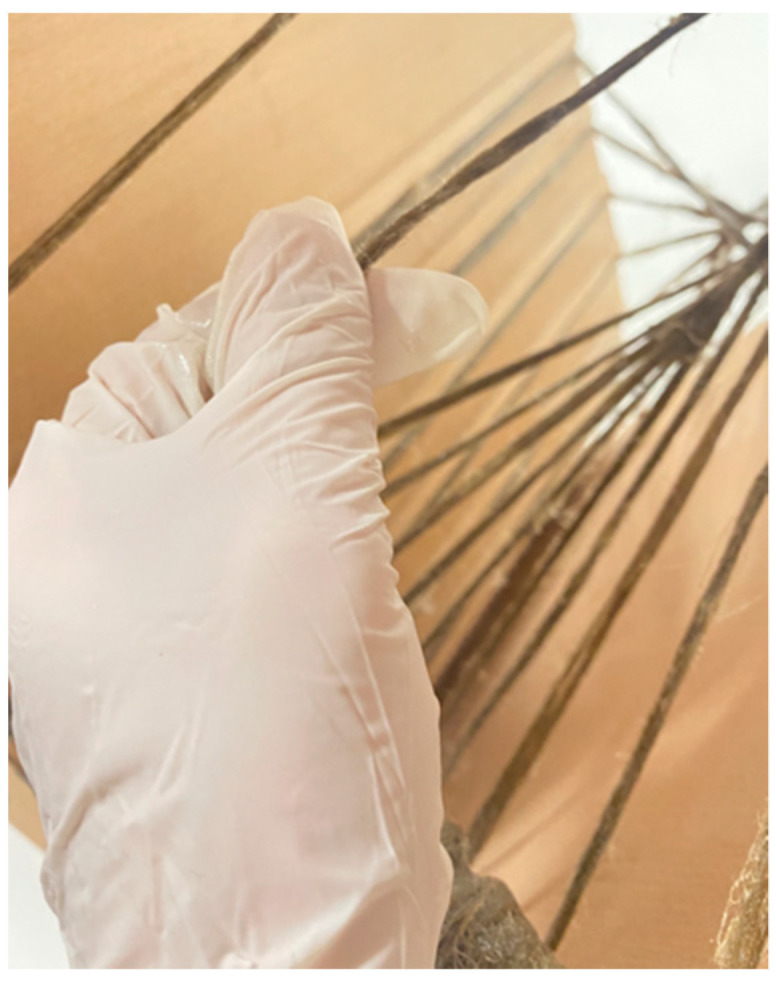
Hand-winding FRP onto embedded timber winding frame for final chair prototype.

Assembly was broken up into two parts. First, the seat was bent into its final position, and in situ lamination was used to fix the ends of the timber surface together, forming the final bent geometry, as seen in Figure 17. The back section of the chair was laminated in place using the same methods. At this stage, the timber frame was very weak and not capable of supporting human loads. In the second assembly step, hand-winding was used to apply the FRP syntax to the embedded timber winding frame, as seen in Figure 18. Finally, the resultant artefact was left to cure at room temperature for 24 h to allow for the flax FRP to reach full structural capacity. 

## 3. Results

**Figure 19:**
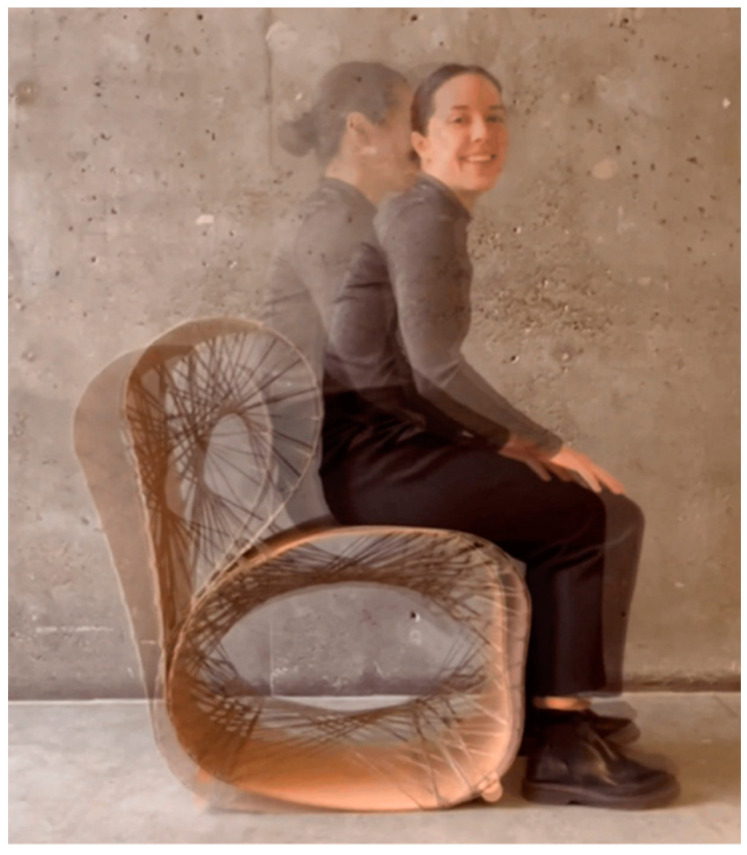
Final rocking chair prototype.

**Figure 20:**
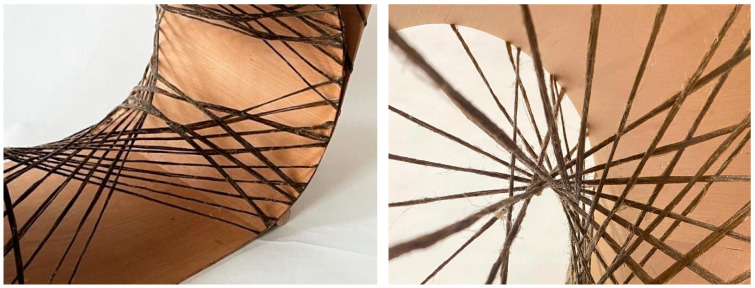
Details of final prototype.

The methods and workflow developed in Section 2 were applied to the production of a final rocking chair prototype, presented in Figure 19 and Figure 20. The prototype demonstrates the use of thin timber veneer in a hybrid system with flax FRP, resulting in a comfortable chair capable of supporting the dynamic loads induced by rocking. Upon completion of the final chair, wooden stoppers were added to the front and back of the ground-facing surface, preventing users from rolling too far forward or backward during rocking. 

**Figure 21:**
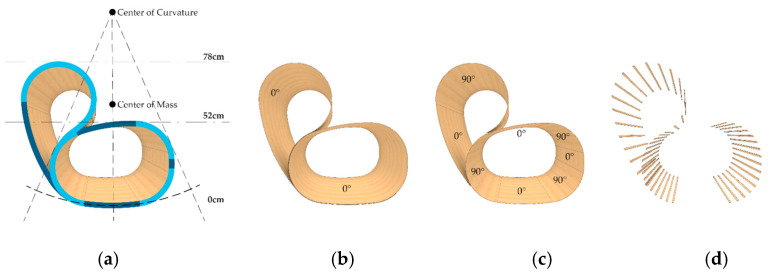
Timber lamination of final prototype: (**a**) complete timber lamination according to the geometric constraints of a rocking chair and timber bending limits; (**b**) first lamination layer with continuous 0-degree veneer; (**c**) second lamination layer with mixed 0-degree and 90-degree veneer; (**d**) laminated anchor teeth.

**Figure 22:**
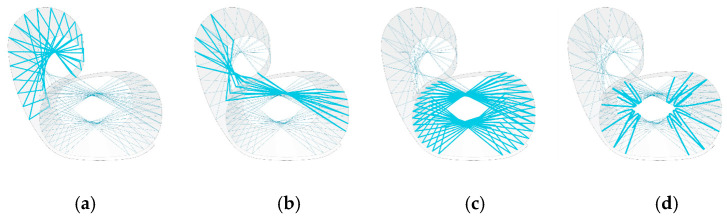
Syntax layers: (**a**) back surface layer; (**b**) bracing layer between seat and back; (**c**) seat surface layer; (**d**) seat bracing layer.

The final prototype used two laminated veneer strips and four syntax layers, as seen in Figure 21 and Figure 22. The seat and back timber elements were flat-laminated with two veneer layers and additive short-edge grooves, then the two layers were laminated in situ to form the final timber geometry and NFRP winding frame. The lamination featured a continuous 0-degree veneer strip as the first layer and mixed 0- and 90-degree segments as the second layer, generating variable curvature in the bent geometry, with radii of 0.2 m in the mixed 0- and 90-degree segments and 0.9 m in the 0 and 0-degree segments. The four syntax layers used alternating cross-edge winding and served different functions in the final performance of the chair. The back surface layer (Figure 22a) and the seat surface layer (Figure 22c) locked the timber geometry in place by following the main deformation regions that would benefit from tensile reinforcement. The bracing syntax layer between the seat and the back (Figure 22b) locked the two timber strips together and prevented delamination. The seat bracing layer (Figure 22d) ensured fibre–fibre interaction and high tension in the seat surface layer.

### 3.1. Material Usage

The total weight of the final prototype was 5.6 kg. Table 1 presents the material usage of beech timber veneer, flax fibre, and epoxy resin individually. The greatest contribution to the total weight and material usage was the beech veneer. Material loss from the subtractive manufacturing of the veneer was not calculated but should be considered when evaluating the overall system waste. 

### 3.2. Prototype Simulation

**Figure 23:**
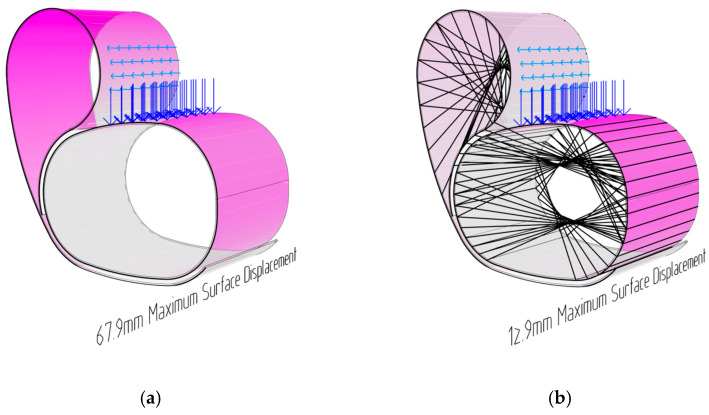
Timber surface displacement finite element analysis: (**a**) the unreinforced model; (**b**) the flax FRP-reinforced model.

To validate the designed flax FRP layup and the proposed timber veneer layer’s thickness, further FEA analysis was conducted. The goal was to minimise the thickness of the timber required, to ensure a mutually reciprocal, hybrid relationship between the two materials, without one material solely handling all the loading forces. Multiple fibre syntaxes and bundle diameters were tested, with primary spans occurring across the green regions indicated above. Figure 23 below highlights the timber surface displacement results when loading unreinforced timber, compared with the flax FRP-reinforced timber. The maximum timber surface displacement was 68 mm in the unreinforced model, compared with 13 mm in the flax FRP-reinforced model. While displacement drastically decreased in the back region of the chair with the presence of flax FRP, the maximum values occurred in the heavily loaded seat. These FEA results supported the initial hypothesis that flax FRP tensile reinforcement placed across the outward bulging deformation regions would stiffen and reinforce the entire material system.

### 3.3. Benchmarking Results

The functional and structural performance of the rocking chair was evaluated through a “seating test” and a “rocking test” with individuals of different weights. The chair supported both seating and rocking, ranging from 55 kg to 100 kg of mass. While less than 1 cm of timber surface displacement occurred when loaded, the maximum values occurred in the seat region, where high forces were applied. This project used a consistent, flax FRP bundle density throughout the entire prototype; however, future iterations would include a denser syntax with a larger bundle diameter to minimise the displacement values where required. No visible deformation or failure was observed in the flax FRP. Destructive compression testing was not performed to determine the maximum load capacity and failure mode of the hybrid system, since only one final prototype was fabricated. The initial design intent aimed to meet the geometric and structural requirements of a functional rocking chair, and minimise material usage through a hybrid composite of thin timber and flax FRP. This intent was met through the final prototype. For long-term durability testing, more prototypes would have to be fabricated, and a more complete set of structural tests should be carried out to achieve results with enough statistical relevance.

## 4. Discussion

The aim of this project was to demonstrate the potential of using thin bent timber as an embedded winding frame for the coreless filament winding of flax fibre–polymer composites (FRPs). Considering both materials as anisotropic, the tailored fibre placement of individual FRP rovings and the fibre directionality of the veneer were important methods for achieving a material hybrid system that is performative and material-efficient. The final prototype, a human-scale rocking chair, demonstrates the potential of using the developed methods towards a material system and fabrication approach capable of being applied to an architectural scale. 

Thin timber veneer was leveraged for its potential for programmed bending to achieve a mould-less fabricated surface that acts as a customised frame for coreless filament winding. The material hybridisation approach allowed two materials that are flexible during fabrication to be combined for the construction of a chair with a very high strength-to-weight ratio. However, unexpected factors were encountered during fabrication. There were high tolerances between the simulations and the results, which did not affect the function of the chair but would need to be explored further if the approach was to be upscaled. 

The in-situ lamination used to join the laminated timber surfaces proved to be a difficult aspect of the fabrication workflow. Holding the timber in the correct position while the wood lamination glue dried required very high accuracy from a manual process, therefore involving a long set-up time of clamps and fasteners. For future explorations, a joinery detail should be included in the timber preparation, such as a dovetail edge-to-edge joint, in order to assist with holding and to provide feedback during fabrication that the correct position has been found. 

Applying coreless filament winding to an embedded flexible frame caused unexpected results during fabrication. Despite only applying hand-wound fibres without extreme tensile forces, the cumulative fibre tensioning throughout the fabrication process induced forces on the embedded timber frame that resulted in unexpected double curvature. This unexpected outcome resulted in the enhanced structural feature of the timber since the double curvature stiffened and strengthened the surface. Future exploration may aim to further explore the potential of intentionally achieving double-curved timber surfaces through similar fabrication methods.

At an architectural scale, the hybrid system has promise in terms of mitigating the reliance of bent timber on a mould or formwork and removing the need for coreless winding projects to utilise a temporary, fabrication-specific frame. The presented approach would allow for differentiation between components without the need for multiple moulds or highly customisable frameworks. The handling of tensile and compressive forces through one of the two materials in a hybrid system is not a novel concept, take, for example, reinforced concrete. However, applying this principle to larger-scale architectural systems of timber and flax FRP, two bio-based materials, is a promising possibility. Upscaling would present unexplored challenges, including a need for high forces required to manipulate timber, streamlining fabrication, and accuracy. However, there is a clear opportunity to exchange many of the manual processes implemented, such as hand winding, for more robust and standardised ones, such as industrial robotic fabrication. 

## Figures and Tables

**Table 1:** Materials used and resulting weight for the final prototype.

Material	Length	Estimated Material Usage	Actual Material Usage
Beech Veneer	8.76 m	3780 g	3780 g
Flax Fibre	155 m	155 g	155 g
Resin Matrix	-	350 g	1100 g
Wooden Dowel Stoppers	-	600 g	600 g
**TOTAL WEIGHT OF PROTOTYPE**			5.6 kg

## Data Availability

Data sharing is not applicable to this article.
